# Gender Differences in Anti-Doping Rule Violations Based on a 19-Year Data Analysis from the Serbian Anti-Doping Agency: National Study

**DOI:** 10.3390/sports13120432

**Published:** 2025-12-04

**Authors:** Gorica Milovanovic, Jelena Rasic Ozegovic, Dejan Jovicic, Nenad Radivojevic, Nemanja Rancic, Jelena Stojicevic, Sonja Marjanović, Zoran Vesic, Milica Vukasinovic-Vesic

**Affiliations:** 1 Anti-Doping Agency of Serbia, 11000 Belgrade, Serbia; gorica.milovanovic@adas.org.rs (G.M.); jelena.rasic@adas.org.rs (J.R.O.); dejan.jovicic@adas.org.rs (D.J.); radivojevicn78@gmail.com (N.R.); 2 Department of Pharmacological Sciences, Medical Faculty of the Military Medical Academy, University of Defense, 11000 Belgrade, Serbia; nece84@hotmail.com; 3 Institute of Hygiene, Medical Faculty of the Military Medical Academy, University of Defense, 11000 Belgrade, Serbia; jelenammaric@gmail.com (J.S.); radakovicvma@gmail.com (S.M.); 4 Department for Social Work and Social Policy, Faculty of Political Sciences, University of Belgrade, 11000 Belgrade, Serbia; zoran.vesic@fpn.bg.ac.rs

**Keywords:** anti-doping rule violations, doping, sports, gender differences

## Abstract

Background: Gender differences in anti-doping rule violations (ADRVs) have been noted in international research, yet systematic analyses are rare. This study addresses that gap by providing the first comprehensive evaluation of the doping prevalence by gender in Serbia. Methods: A 19-year retrospective quantitative analysis was conducted on data collected by the Anti-Doping Agency of Serbia (ADAS) between 2006 and 2024. In total, 14,919 doping controls were performed, including 10,912 (73.11%) on male athletes and 4007 (26.89%) on female athletes. Results: Across this period, 146 ADRVs were identified, with a clear gender imbalance: 128 (87.32%) cases involved male athletes and 18 (12.68%) female athletes. A Chi-Square test confirmed a significant association between gender and ADRVs, χ^2^(1, N = 14,919) = 15.11, *p* < 0.001, indicating that male athletes were more likely to violate anti-doping rules. Substance profiles also differed: anabolic agents (S1) dominated overall, while stimulants (S6) and cannabinoids (S8) were more frequent in males, and diuretics (S5) and hormone modulators (S4) in females. Conclusions: These findings reveal a pronounced gender disparity in doping behavior and substance choice, providing a foundation for further research and emphasizing the need for gender-sensitive anti-doping education and policy.

## 1. Introduction

The term “doping” in the World Anti-Doping Code (WADC) does not refer to a singular act but rather is based on the occurrence of one or more anti-doping rule violations (ADRVs). There are 11 officially recognized ADRVs, encompassing a broad spectrum of prohibited conduct, including the presence, use, or attempted use of banned substances or methods; evasion, refusal, or failure to submit to doping control procedures; failure to submit whereabouts; tampering or attempted tampering with any aspect of the doping control process; possession, trafficking, or attempted trafficking of prohibited substances or methods; administration or attempted administration of such substances or in- or out-of-competition methods; complicity in anti-doping rule violations; engagement in prohibited association with individuals serving sanctions; and acts intended to intimidate, discourage, or retaliate against individuals for reporting doping-related misconduct [1]. This model seeks to ensure consistency and harmonization in the implementation of anti-doping policy across all sports and nations globally.

An Adverse Analytical Finding (AAF), which may also be referred to as a positive result, is a report issued by a WADA-accredited laboratory indicating the presence of a prohibited substance and/or its metabolites or markers, including evidence, found in an athlete’s sample, of the use of a prohibited method. Operationally, an AAF is produced only after a full analytical workflow, which consists of initial screening followed by confirmatory analysis, has been completed using validated methods, in accordance with WADA Technical Documents and decision limits. This applies to urine, blood/serum, and any other sample matrices permitted under the International Standard for Laboratories. AAFs were identified in 0.80% of the samples analyzed in 2023, representing a slight increase in rate compared to the previous year (0.77%) [2]. These findings sit alongside WADA’s periodic ADRV reports, which consistently show that, worldwide, only a small fraction of all tests lead to ADRVs and that men account for most sanctions. By comparing our national data against these global benchmarks, we can determine whether the gender gap and substance profiles observed in Serbia align with or diverge from broader international patterns. It is very important that an AAF itself is not an anti-doping rule violation (ADRV), but rather the stepping stone to results management. Then, several protections mechanisms apply, including informing the athlete that disciplinary proceedings have been initiated and advising them of their right to have their B-sample analyzed. Additionally, any possibility of Therapeutic Use Exemptions (TUEs) should be considered, the sample integrity and chain-of-custody should be investigated, and all procedures should also be considered, before any ADRV is asserted. Related but different outcomes are Atypical Findings (ATFs) that do not reach the level of an AAF but raise a credible suspicion or call for further targeted follow-up testing to examine the possibility that such a finding may have resulted from something other than doping, such as normal physiological variation, therapeutic use, or sample tampering. Laboratories also take into consideration variables such as urine specific gravity (for correction of diluted specimens) and endogenous production (for, e.g., steroid ratios and isotope ratios), among other examples, to reduce the chance of a false positive result. While AAFs are not equivalent to an ADRV, they constitute a sensitive early-warning indicator of potential doping that, when aggregated over time, represent a powerful intelligence signal. There are longitudinal trends in AAFs helping anti-doping organizations calibrate the risk-based test distribution and focus on educational efforts, as well as identify sports or periods of the season that carry an elevated risk. Also, because samples can be retained and re-examined as the testing technique advances, according to the limits defined by the Code, an AAF today, or an ATF that triggers long-term monitoring, may constitute evidence that can support the resolution of a future case [1].

There is substantial evidence regarding the adverse effects of the use of doping substances. The consumption of anabolic–androgenic steroids is correlated with cardiomyopathy, sudden death, hypertension, and arrhythmias. Among long-term users, the risks of developing a cardiovascular disease and premature death are significantly increased [3,4]. Beyond clinical events, cardiac imaging and pathology consistently show concentric left-ventricular hypertrophy, impaired systolic/diastolic function, and myocardial fibrosis in chronic users, often accompanied by atherogenic lipid profiles and endothelial dysfunction [5]. Recent studies involving coronary CT imaging in recreational athletes have also associated cumulative AAS exposure with a higher plaque burden and myocardial changes [6]. The use of AAS also has neuroendocrine effects. Prolonged suppression of the hypothalamic–pituitary–gonadal axis, reduced spermatogenesis and azoospermia in men, and virilization with menstrual disturbance in women have been repeatedly documented [7]. In addition to somatic effects, there is evidence that people who use anabolic–androgenic steroids can experience long-term aggressive behavior, depressive episodes, and feelings of dependence. Meta-analyses and a clinical review have shown increased risks of depression, anxiety, and cognitive consequences in chronic users [8]. Randomized data and pooled analyses also show a measurable increase in self-reported aggression after AAS exposure, and dependence syndromes occur in a substantial minority of users. Administration of recombinant human erythropoietin elevates hematocrit and blood viscosity and puts patients at a higher risk of thrombosis, myocardial infarction, or stroke [9]. This risk is consistent with meta-analyses of erythropoiesis-stimulating agents in clinical populations, showing increased venous thromboembolism, stroke, and hypertension, with dose and hemoglobin targets acting as key modifiers [10]. While stimulant abuse may result in acutely increased alertness, this also carries risks, including hypertension, arrhythmia, hyperthermia, and the potential for developing drug dependence [11]. When athletes train hard, especially in warm environments with a high temperature and humidity, studies demonstrate that agents such as modafinil and amphetamine derivatives elevate their core temperature and heart rate, thereby increasing the risk of heat illness and arrhythmias. The risk is often compounded by “stacking” and ancillary drug use (e.g., SERMs, aromatase inhibitors, hCG, diuretics, thyroid hormones), which introduce additional cardiovascular, hepatic, and psychiatric liabilities and complicate recovery. Besides adverse health effects, athletes may face sanctions, disqualification of results, reputational damage, and career interruption once they have committed ADRVs [12,13].

The literature on gender differences in doping is scarce, but several recent studies have offered valuable information on the issue. A systematic review of multiple studies found that the current evidence is insufficient to draw firm conclusions about gender-related doping trends; however, it does highlight certain context-specific gender doping patterns [14]. Outside elite sport, reviews note that AAS use in women is generally low in the wider population but notably higher in bodybuilding/gym subcultures. These findings underscore the need for caution when extrapolating patterns observed in elite testing to broader community settings [15].

Nevertheless, most of the evidence is derived from multi-country composite analyses and individual-sport cohorts or survey-based samples, with longitudinal national datasets collated by smaller national anti-doping organizations (NADOs) being scarce. Therefore, Serbia serves as a good example regarding several aspects of sporting activity, such as strong involvement in team/case and combat sports, close to a visible bodybuilding and powerlifting scene, and limited resources compared to other Western European national anti-doping organizations. This is also typical because a high percentage of athletes compete in both domestic and international competitions. A national review of ADRVs from ADAS thus extends international WADA reports by presenting sex-separated data within a single, mid-size European testing program.

Studies among young elite athletes based on survey data suggest very pronounced gender differences in doping use, as measured by self-reports. It is reported that in certain subpopulations, the gap in doping prevalence between males and females narrows [16], so it may be that different sociocultural expectations moderate this relationship. Indirect methods of questioning provide higher estimates than direct self-report, and in some samples, there is less sex difference, indicating that a reporting bias and sport-mix effects should be considered when studying “gender gaps” [17]. Among these subsets, both supplement use and use of dietary supplements typically co-vary based on the regulations of a supplier and the attitudes related to doping during a competitive season, suggesting an athlete’s trajectory from experimentation with supplements to pro-doping attitudes [18]. The literature also highlights concerns about anti-doping literacy, particularly among athletes. For instance, women may be at increased risk of unintended doping due to ineffective educational aids [19]. Qualitative and survey data among adolescent athletes argue for restricted, late, or fragmented access to structured anti-doping education, particularly before the mid-teens [20]. These gaps overlap with real-world dangers: contamination of supplements is still widespread, providing a path for accidental positives where there is a lack of certification and vetting [21]. In this context, the finding that women may be at greater risk of accidental doping in some contexts (due to different product choices, patterns of medicalization, or reduced access to targeted advice) is consistent with education gaps highlighted in athlete surveys [18]. A comprehensive review by national anti-doping organizations is therefore essential to map gender-specific trends using suitable denominators, harmonize sport-specific exposure data, and align education audits with outcome information [22]. Priorities should include sex-disaggregated monitoring along youth pathways, among-sport (rather than crude pooled) comparisons, and routine monitoring of supplement-use sources as well as the use of certified supplement batches.

Anti-doping education plays a key role in the prevention of the use of doping agents among athletes, and there is evidence that it may promote more positive attitudes towards doping prevention [23]. Randomized and quasi-experimental studies report short-term reductions in favorable attitudes after targeted education, delivered in person, online, or using some other tools, with some decay over time, which supports the use of booster sessions and season-long sequencing [24]. The International Standard for Education prepared by WADA should be applied, respecting both value-based and risk-based contents. Athletes’ early exposure and cohort-specific tailoring in the process of education are of crucial importance [25]. Values-focused interventions with elite athletes who are adolescents show improvements in susceptibility and moral disengagement measures, suggesting that teaching decision-making and norms can complement rule-based modules [26]. Understanding how gender influences the effectiveness of these educational initiatives is essential for devising programs that more accurately address the needs of different groups and resonate with those groups. Sex-disaggregated planning is warranted because exposure, the sport mix, injury care, and supplement behaviors differ across women’s and men’s pathways; these differences shape what content is salient and when it should be delivered [27].

The Anti-Doping Agency of Serbia (ADAS) has been compliant with the World Anti-Doping Code since its establishment. Its work is guided by principles of transparency, independence, data protection, reliability, objectivity, effectiveness, and cooperation with the wider public as well as with state institutions and national and international sport bodies. These principles are reflected in the planning of testing, the procedure of sample collection, the protection of athlete information, and the communication of results to stakeholders. Over the past two decades, ADAS has collected thousands of samples in competition and out of competition. Samples have been analyzed at the WADA-accredited laboratory in Seibersdorf, Austria, following the applicable international standards and technical documents. Procedures include risk-based test distribution planning, a secure chain of custody, the use of the ADAMS system for data management, and results management in line with the Code. Both urine and blood samples are collected when appropriate, and biological passport information is maintained where required. The overall aim is to protect athlete health and preserve fair play in sport through reliable detection and consistent adjudication.

The current study uses this multi-year dataset to describe patterns in adverse analytical findings and anti-doping rule violations, with a specific focus on differences by sex. Outcomes are examined by substance class, sport category, and testing context, and rates are interpreted with attention to exposure to testing and the composition of tested populations. This method enables the study to go beyond crude counts and determine whether such sex differences observed are reflective of actual variation in exposure rather than sampling or the event mix. To our knowledge, this is the first study examining the gender-specific ADRV pattern based on completed 19-year testing data from a single national anti-doping agency in Southeastern Europe, which simultaneously interlinks substance classes, sport categories, and testing exposure through one national dataset.

## 2. Materials and Methods

This is a 19-year retrospective longitudinal analysis of anti-doping data collected by ADAS (Belgrade, Serbia) (from 2006 to 2024). The first task was to investigate gender differences in ADRVs by analyzing the distribution of doping controls and confirmed violations among male and female athletes across all tested sports in Serbia.

The source of the data for all recordings is the ADAS official testing records. The dataset contains data on the overall number of doping controls performed per year, athletes’ sex, and the actual number of confirmed ADRVs. However, the dataset is limited to athletes tested by ADAS, and the sample represents only athletes who had been exposed to testing. The doping controls were performed both in-competition and out-of-competition. In this analysis, only the subsequent features representing the following variables were extracted and manipulated:

- Total number of doping controls per year;
- Gender of athletes tested;
- Total ADRVs detected between male and female athletes.

Personal data, including athletes’ names, birth dates, or clubs are absent in this study. The data was summarized at the group level (male and female athletes) to ensure subject confidentiality.

The study sample consisted of all athletes sampled by the Anti-Doping Agency of Serbia from 1 January 2006 to 31 December 2024. During this time, a total of 14,919 doping controls were performed. In total, 26.89% of doping procedures were performed on female athletes, while the rest of the procedures were performed on male athletes. The controls included athletes from numerous individual and team sports.

ADRVs were defined operationally as all cases in which results management concluded that an athlete had committed at least one anti-doping rule violation under the World Anti-Doping Code in force at the time of the sample collection. During the study period, ADAS operated under the 2003 Code (effective for our data from 2006 to 2008), the 2009 Code (2009–2014), the 2015 Code (2015–2020) and the 2021 Code (2021–2024). In practice, almost all cases in this dataset arose from Article 2.1 (‘presence of a prohibited substance or its metabolites or markers in an athlete’s sample’) following an Adverse Analytical Finding; non-analytical ADRVs (e.g., whereabouts failures, tampering, trafficking, or prohibited association) were rare and are included in the overall counts but were not analyzed separately by type due to their small number. For uniformity, only the cases that have been finally processed are being considered. All the data was validated manually for both consistency and completeness prior to analysis.

For substance-level analyses, each prohibited substance was coded according to its class on the WADA Prohibited List in force at the time of sample collection (S1–S9 and P1). For reporting in this paper, we aggregated at the class level (e.g., S1 Anabolic Agents, S5 Diuretics and Masking Agents). 

Statistical analyses were conducted with IBM SPSS Statistics v. 23. A *p* < 0.05 level was used as an indicator of statistical significance. The data is presented as frequencies of individual categories. The Chi-Square test was used as the primary method to compare categorical variables between groups. Additionally, multivariate analysis was conducted using logistic binary regression and interpreting the B coefficient, Nagelkerke’s R^2^, and the *p* value of the model.

## 3. Results

The number of doping controls during the 19 years between 2006 and 2024 was 14,919 controls. The sex distribution of these controls shows a strong female–male imbalance, with 10,912 (73.11%) male tests and 4007 (26.89) female tests carried out. A total of 146 ADRVs were observed. The majority of these violations were conducted by male athletes, constituting 128 violations of the 146 (87.32%). However, females committed 18 ADRVs (12.68%) for 26.89% of the total number of athletes analyzed (Table 1). As a percentage of tests, this resulted in an ADRV rate of 1.17% in sampled male athletes (128 ADRVs/10,912 controls) and 0.45% for sampled female athletes (18 ADRVs/4007 controls), showing that men were about 2.6 times more likely than women to commit an ADRV even after adjusting for the fact that more tests were performed on male competitors.

Our study showed a statistically significant difference in anti-doping rule violations (ADRVs) according to gender χ^2^ (1, N = 14,919) = 15.11, *p* < 0.001, indicating that male athletes showed higher rates of ADRVs than expected based on the proportion of testing (male vs. female: 87.32% vs. 12.68%) (Table 1). This result suggests that ADRVs’ frequency distribution by gender is not a random effect and that there is a statistically significant association between the gender of an athlete and their odds of being found to have committed a doping rule violation.

A further examination of the categories of doping substances revealed in the adjudicated cases may help to better understand gender disparities in AAF. The findings present characteristic styles of using and the substance classes and types used by male and female athletes in Serbia. Categories, numbers, and fractions of substances are listed in Table 2 by gender. Summary totals from these tables are based on the total number of substances associated with all breaches of the AAF and may at times exceed the total number of AAFs, even if a single AAF involved more than one substance or method.

The fact that the data in Table 2 is categorical justifies the utilization of the Chi-Square test as an indication of statistical significance. The Chi-Square test showed that there was statistical significance in the S4 (Hormone and Metabolic Modulators) and S5 (Diuretics and Masking Agents) classes, as the *p* values were 0.002 and 0.004, respectively. The significance level through which the values were analyzed was *p* < 0.05. S1 (Anabolic Agents) and S8 (Cannabinoids) were not statistically significant in terms of their *p* values, and the significance level taken for statistical analysis. Chi-Square tests were not performed for categories with zero counts due to a violation of minimum expected frequency assumptions.

Binary logistic regression was performed, where gender was considered as the dependent variable and the type of sport (individual/team) was selected as the covariate. Nagelkerke’s R^2^ was 0.094, indicating that the type of sport explains 9% of the variance in gender, which is considered a small effect. The B coefficient (B = −2.154) signifies that team sport athletes are less likely to be female compared to individual sports. The model is statistically significant at 0.039, showing that the sport type predicts the gender of doping-positive athletes. The model classifies 86% of cases overall; however, this can be explained by the high number of males in the sample. Other covariates (e.g., competition level, testing frequency) were considered but excluded.

The data shows S1 (Anabolic Agents) to be the most common category of substance misuse, representing 47.59% and 54.28% of male and female athletes’ violations, respectively. Differences remain substantial in the other categories. S6 (Stimulants) and S8 (Cannabinoids) are the next most reported substance abuse violations in male athletes, at 22.29% and 15.06%, respectively. On the contrary, in female athletes, S5 (Diuretics and Masking Agents) is the second highest category with 31.43%, followed by S4 (Hormones and Metabolic Modulators) with 8.57%. These results are consistent with other research supporting gender-specific substance use profiles.

Among female athletes, substances from Group S1 (Anabolic Agents) were detected in nine cases—seven of them in bodybuilding (77.78%), one in boxing, and one in volleyball. In contrast, among male athletes, S1 substances were found across 18 different sports. The most common sport was bodybuilding with 23 out of 67 violations (34.32%), followed by powerlifting (n = 8 or 11.94%), kickboxing (n = 6 or 8.96%), and boxing (n = 5 or 7.46%). These patterns indicate that among women, S1 ADRVs are concentrated in sports where muscularity and leanness are explicitly rewarded (bodybuilding and combat sports), whereas in men, S1 violations span a broader array of power and combat sports (bodybuilding, powerlifting, kickboxing, boxing, and others), reflecting wider performance goals such as increased strength, speed, and recovery. However, our study shows that there is a different pattern in using anabolic agents between female and male athletes.

## 4. Discussion

ADAS’s dataset from 19 years of work provides rare longitudinal insight, useful for examining gender patterns in anti-doping outcomes within a single national system and a whole range of different sport disciplines. The length of follow-up and completeness of the national testing pool across this entire period constitute a key methodological strength of this study, allowing us to examine relatively stable gender patterns despite multiple revisions of the WADA Code and Prohibited List. Our main result, which shows that male athletes are significantly more likely to commit an ADRV than female athletes, corresponds to global evidence showing lower female AAF/ADRV rates [28]. Moreover, our study’s results correspond to not only the overall results of other studies but also the magnitude of the male–female gap common to multi-country analyses, suggesting that upstream determinants (e.g., sport culture, risk perception, substance market structure) may be operating similarly across various contexts [28,29].

Empirical studies show striking discrepancies regarding incidents of ADRVs by sex, as reports at the national level provide evidence that AAFs in women are lower in comparison to men, particularly for anabolic agents. In contrast, some drugs (such as β2-agonists and diuretics) have been reported more often in female test-positive athletes [30]. Recent global figures align with this pattern, and among ADRVs arising from AAFs in 2022, 77% involved men and 23% women [31]. Integration of the doping data further reinforces these conclusions and provides evidence that, while anabolic agents are the most detected drug in a broad cross-section of sports, their occurrence differs substantially between the genders and different sports [32]. A significant difference in the proportion of monitored substance classes between sexes was reported by one study. The reported use of substances (generally) from classes S1 (Anabolic Agents), S4 (Hormone and Metabolic Modulators), and S8 (Cannabinoids) was lower among female athletes than their male counterparts and higher from classes S3 (Beta-2 Agonists), S5 (Diuretics and Masking Agents), and S9 (Glucocorticoids). This study also found that, for two substance classes, women only used recombinant erythropoietin (S2), while men used more substances, including hGH and growth factors. In the S4 class, anti-estrogens were the only substances detected in women; however, men used aromatase inhibitors and metabolic modulators. Comparable class-specific patterns are reported in a seven-year national dataset (2013–2019), where women showed a higher relative detection of terbutaline (S3) and furosemide (S5), while men showed clustered use of S1 combined with S4 agents and broader S2 profiles; the same study also documents that women accounted for about 22% of all controls over that period, underscoring the need to report sex-disaggregated denominators [33].

In addition to differences in overall prevalence rates, the distribution of prohibited substance classes exhibits notable gender-specific patterns. Among female athletes, anabolic agents (S1) prevail but are heavily concentrated in bodybuilding, which emphasizes muscularity and leanness as scoreable standards. This focus is supported by recent literature that has linked sport types emphasizing physique to increase steroid exposure among female athletes [30]. Our national data therefore suggest that for Serbian women, ADRVs are primarily linked to physique-oriented and weight-class sports, while for men, ADRVs also encompass team and strength/power sports where anabolic agents and stimulants are used to support the training load and explosiveness, and, in some cases, for recreational use (e.g., cannabinoids). This supports the idea that performance requirements and body ideals in sports steer athletes toward certain substances. Also, men are more likely to test positive for S6 (23.3%) and S8 (21.3%), whilst women show higher rates of detection of diuretics/masking agents (S5) and hormone/metabolic modulators (S4). Data collected from international surveillance has also indicated that men are more likely to be found with substances linked with acute performance enhancement or recreational use, whereas females are commonly found with drugs linked to body-mass control and endocrine function [34,35]. These class-specific patterns suggest that ADRVs represent a spectrum of behaviors rather than a single phenomenon. The findings in our study show similarities but also notable differences when comparing with reports from Western European settings. Consistent with some studies, male athletes show a predominance of S1 violations with additional clustering of stimulants and cannabinoids [30,33,34,35]. However, unlike some Western European datasets where S3 (β2-agonists) and S5 (diuretics) dominate among female test-positive athletes, Serbian female ADRVs remain heavily anchored in S1, with diuretics and hormone modulators in a secondary position. This suggests that, in our context, female doping behaviors may be more tightly coupled to bodybuilding and strength-aesthetic sports than has been described in some larger Western European programs.

These sex differences may be related to the interaction of biologic, sociocultural, and sports-specific factors. The observed gender gap is likely shaped by at least three sets of external factors that are themselves gender-biased: (i) sport participation and testing exposure, (ii) sport-specific performance demands and body ideals, and (iii) broader sociocultural expectations about masculinity, femininity, and risk-taking. Consider androgenic-anabolic agents with strong sexual dimorphism in their pharmacodynamics, and adverse event profiles (including virilization risk, which may discourage use in some women), but larger hypertrophic effects for athletes with less muscle mass at a given dose. These might paradoxically elevate demand in regard to the female physique depending on the sport and if there is a heavy emphasis on the visible hypertrophic/optical appearance of necessarily muscular bodies [36]. In social terms, some cultural ideals of masculinity/femininity and styles of coaching may work to encourage or restrain risk-taking in relation to doping within a certain environment. Sport discipline prejudices are also a significant factor. The pressures on weight-class and aesthetic sports, driven by high expectations, increase athletes’ attraction to S4/S5 agents, whereas in power/speed sports, the anabolic trajectory dominates, making S1 substances more dominant; meanwhile, as this combination input is exaggerated across these types by a gendered participation gap, additional burdening constraints are increased or distorted. Economic rewards and exposure play a role as well, since the larger the number of men in professionalized leagues, the more commercially attractive and viewed the sport becomes. This also matters when it comes to discovery dynamics and therefore experimentation decisions. Taken together, these factors help to explain why, even when the test volume is considered, Serbian male athletes exhibit a substantially higher ADRV rate than female athletes, and why the substance-class profiles differ across sexes.

When examining the “sport type” of doping-positive athletes, the sport type emerged as a statistically significant (yet modest) predictor, since team sport violations were less likely to be associated with female than male athlete offenders, such as individual sport offences (Nagelkerke R^2^= 0.094), showing that only a small portion of the variance was explained by the discipline. The regression model explains very little and itself suggests that future studies need more multivariate analyses. However, this tendency is in general agreement with large anti-doping databases and survey research, which shows that men are more likely to use or report using performance-enhancing agents than women both at a general level and also nationally [14]. Moreover, research has revealed moderate significant effects of the sex on the type of sport and doping interaction, where male athletes in both team and individual sports used performance-enhancing substances at higher rates than female ones, and where male players had higher scores for their likelihood of doping (e.g., basketball and handball) [37]. When all party-level variables are taken together, these findings imply that the sport type on its own contributes relatively little to the prediction, but the gendered pattern we found in doping-positive cases matches with previous findings in the literature describing male athletes—especially those who engage in team sports—as a more risk-laden group for doping.

Such findings have practical implications for prevention and testing. Education must be both sport- and gender-inclusive in practice. For women in physique- or weight-class-restricted sports, such curricula should be dedicated to educating on the potential health and detection ramifications of S1 use; the endocrine and renal effects of S4/S5 strategies; as well as using evidence-based non-pharmacological approaches to body periodization. Men: An additional focus is needed on team and combat sports where there is a high likelihood of stimulant abuse, polydrug effects with pseudoephedrine, under the influence of fatigue, and spillover risks from social use of cannabis or synthetic cannabinoids, where anti-doping rules do not align with the civil law. The testing plan can involve gendered and sport-specific risk indicators without sliding into discriminatory practice, such as targeted windows around known weight-cut periods, in-season peaks in stimulant temptation, or off-season cycles in physique sports, all while maintaining proportionality and respect for athletes’ rights. Those working as supporting staff for athletes should be trained to identify gender-specific red flags and provide safe alternatives.

Despite its benefits, this study has several limitations. It is a retrospective, quantitative cross-sectional study of previously reported ADAS data. While it can map trends and associations, the nature of our study is such that it cannot resolve the causal pathways linking motives, contexts, and substance choices. Across 19 years, multiple moving parts changed: the WADA Prohibited List changed and analytical technologies improved. Each shift modifies both the true underlying prevalence and the probability of detection, making trend analysis sometimes challenging. The ABP (hematological and steroidal modules) further reweighted detection towards certain classes and usage patterns, potentially affecting gender comparisons if ABP penetration differed by sport where one gender predominates. Moreover, different ADRVs may vary by gender and sport but are not decomposed here. Procedural elements and behavioural factors could introduce differential verification probabilities.

The sport composition of the sample is important: If women are overrepresented in sports where S1 use is at once more advantageous and surveilled (e.g., bodybuilding competitions with high surveillance), proportions by class might reflect sport ecology rather than gender per se. There is also the potential for age and an athlete’s career stage to be confounding variables, with differences in debut age and peak performance windows by sport (and sex), where younger cohorts might have different risk appetites or supplement practices. Access to medical oversight and the quality of nutritional counseling may also vary by sex and sport, which would impact both the decision-making quality and TUE use for valid medical conditions that overlap with S4 agents (e.g., selective estrogen receptor modulators following injury).

Equity and ethical concerns are very important. There is a clear need for gender-responsive prevention efforts to reduce incentives and chances for the use of doping substances. A “one-size-fits-all” message is not appropriate in this context, but there is also a need to be cautious in transmitting the appropriate message so that gender stereotypes are not being reinforced. For example, regarding “weight-control agents” as health-endangering rather than an existential failure can have more of an effective appeal among women participating in aesthetic and weight-class sports, whereas interventions targeting coping/anxiety and sleep management can reduce stimulant and cannabis temptations for men without further stigmatizing help-seeking. It is essential to guarantee that, in male and female programs, sanctioning, access to education, and medical supervision are equivalent between genders, in order to prevent structural drivers of inequality.

For policy and practice, three courses of action are identified. First, incorporate mixed methods into normal surveillance: coupling sport-stratified quantitative tracking of these outcomes with confidential interviews and validated psychometrics on motives, risk perception, and team norms can reveal decision pathways otherwise opaque in laboratory data. Second, further develop risk-based test planning with dynamic indicators: competition schedules, known weight-cut durations, cycles of training camp periods, and transfer/contract windows where performance anxiety skyrockets. Third, fund ASP capacity-building to limit accidental violations, especially S4/S5 exposure from inadequately vetted supplements or off-label prescribing; structured checklists and decision aids tailored for sport and sex can minimize mistakes.

Subsequent research should move beyond the current focus of between-gender heterogeneity to examine within-gender heterogeneity. The clustering of female S1 findings in one sport indicates that the findings should not be overgeneralized; micro-epidemics can prevail within a competitive circuit. Associating the ADRV timing with training mesocycles, travel loads, and injury episodes may uncover windows of increased vulnerability. Longitudinal cohorts that follow athletes with year-over-year observation for junior-to-senior transition may help to disentangle changes by sex in norms and incentives. From a methodological standpoint, reconciling denominators and accounting for list/technology changes would improve inference around actual behavioral trends. Theme-wise, we need to look further at the motives: cultural scripts around female/masculine identity and compliance, body image pressures, and financial insecurity may well mediate perceived benefits/harms in a way not covered by the biological rationale alone. Third, the inclusion of evaluation components in context-specific programming can provide causal evidence of which messages change intentions and by which delivery mechanisms men and women are best reached.

Finally, this study of ADAS 19-year records points to a stable gender gap in ADRVs. This gap is consistent with international studies and can be plausibly explained by intertwined biological, social, and sport-ecological factors. Such complexity directs is to adopt a precise prevention approach rather than generic one, and that approach must be aimed at protecting health and fairness while reducing unintended harm.

## 5. Conclusions

This report offers strong evidence of the extraordinary male-to-female ratio of anti-doping rule violations in Serbia during the last 19 years. The results furthermore establish that male athletes are significantly more likely to commit ADRVs than female athletes and illustrate that prohibited substances are used differently for each gender. The present work lays the groundwork for further refined inquiry into relatively subtle matters to assist in driving more effective and gender-responsive anti-doping policymaking and education in the future. Practically, this suggests that targeted anti-doping education and risk-based testing in sports with high male ADRV rates (particularly strength, combat, and team sports) should be focused upon, as well as the female sport disciplines where S1, S4, and S5 detections are concentrated. Health interventions to prevent substance misuse should specifically target sex-specific risk factors (e.g., pressure on body image in aesthetic and weight-class sports, stimulant or cannabis use around high-pressure competition periods in team sports), ensuring that both sexes have equal access to quality information and optimal medical care. Finally, if ADAS can integrate sex-disaggregated monitoring into routine test allocation planning, then the organization can follow up on interventions and adjust its priorities in real time.

## Figures and Tables

**Table 1:** Summary of doping controls and anti-doping rule violations by gender (2006–2024).

Category	Total	Number of Male Athletes	Number of Female Athletes	Male % of Total	Female % of Total
Doping Controls	14,919	10,912	4007	73.11%	26.89%
Anti-Doping Rule Violations	146	128	18	87.32%	12.68%

**Table 2:** Number of prohibited substances by class present in AAF in both genders.

Prohibited Substance Class	Male	Female	Sig. (*p* Value)
S1. Anabolic Agents	79 (47.59%)	19 (54.28%)	0.471
S2. Peptide Hormones, Growth Factors, Related Substances, and Mimetics	2 (1.21%)	0 (0.00%)	
S3. Beta-2 Agonists	1 (0.60%)	0 (0.00%)	
S4. Hormone and Metabolic Modulators	1 (0.60%)	3 (8.57%)	0.002
S5. Diuretics and Masking Agents	20 (12.04%)	11 (31.43%)	0.004
S6. Stimulants	37 (22.29%)	0 (0.00%)	
S7. Narcotics	1 (0.60%)	0 (0.00%)	
S8. Cannabinoids	25 (15.07%)	1 (2.86%)	0.051
S9. Glucocorticoids	0 (0.00%)	0 (0.00%)	
P1. Beta-Blockers	0 (0.00%)	1 (2.86%)	

## Data Availability

The original contributions presented in the study are included in the article, further inquiries can be directed to the corresponding author.

## Abbreviations

The following abbreviations are used in this manuscript:

WADC	World Anti-Doping Code
ADRVs	Anti-Doping Rule Violations
AAF	Adverse Analytical Findings
WADA	World Anti-Doping Agency
ADAS	Anti-Doping Agency of Serbia
